# Supplementary material for: Low LEF1 expression is a biomarker of early T-cell precursor, an aggressive subtype of T-cell lymphoblastic leukemia
Source: PLoS One. 2020 May 13;15(5):e0232520. doi: 10.1371/journal.pone.0232520 (PMC7219738; doi:10.1371/journal.pone.0232520)
Supplement: Attachment — Submitted filename: Response_to_Reviewers.docx [file pone.0232520.s006.docx]

Thanks for the reviewers’ advice. The manuscript was edited accordingly.

Reviewer #1: The authors considerably improved their paper. I am not competent enough to address the biostatistical issues related to the manuscript; here I fully rely on the opinion of referee 2. I still think that the discussion on the role of LEF1 in tumorigenesis, which is textbook knowledge, does not add anything to the message of the paper and should be either shortened or omitted.

Thanks for reviewing our manuscript. Considering the reviewer's suggestion, we tried to shorten the discussion on LEF1's biological function, but found that each sentence is supporting our hypothesis and point of view. In addition, the description and discussion based on re-analyzing the TCGA Pancancer data is our original work, not referred from any textbook or other publications. We prefer to keep them. But if the reviewer has a more specific suggestion, e.g. removing a specific sentence, we could try to revise this section again.

Reviewer #2: Wang and Zhang in their revised research article entitled: “low LEF1 expression is a biomarker of early T-cell precursor, an aggressive subtype of T-cell lymphoblastic leukemia” have addressed most of the suggestions raised. However, I think that many minor changes are required to make the manuscript suitable for the publication and I encourage the author to do them. I appreciated the availability of the scripts used.

Minor points above:

In the title please indicate the first letter in upper case.

Corrected.

Line 68 reformulate the sentence, indeed “whole transcriptome and by the 35-gene ETP signature were conducted respectively on...” may be understood as one analysis was applied on TARGET while the other on GSE42328 instead both the analyses were applied to both the data sets.

“respectively” is deleted.

Lines 109-110 explain the concept of specificity and sensitivity and how you calculated them adding few sentences on the methods, maybe in the “Other Statistic section”.

Explained as suggested.

Lines 154-163 please substitute the word “study” with synonyms.

“study” is replaced with “publication”.

If, from TCGA dataset, the authors used RNA sequencing data, it would be useful to add the number of genes (if specified) for completeness.

We only used the expression level of LEF1 gene from the RNA sequencing dataset.

Unsupervised clustering section: the authors have deleted the selection of the 5,000 genes which was reported in the previous version of the manuscript; in my opinion it is important to add it back in order to let the reader fully understand the method used to select these genes.

Thanks for reminding. It was removed by mistake. That sentence is added back in this revision.

Supervised prediction section, please add a reference in the first two sentences of this paragraph. Moreover, please provide the numbers and parameters used as pointed out in the answer to the reviewer. Finally I do not see along the text that the GSE42328 data set is called neither “external” nor “secondary” (as indicated in the answer to reviewers) but only “validation”, is it intended?

Reference is added and numbers are provided.

To be consistent and concise, we decided to call the GSE42328 the validation dataset.

Please, add in the m&m, under the section “Other Statistics”, the acronym DEGs when referring to the explanation of this methodolgy. Moreover, please add a reference and the method used to do the Cox regression and add the “survival” R library (with its citation) if it was used to calculate the survival rate.

Added as suggested.

Supplementary Tables and Materials need captions (legend) to explain what they show. I suggest merging all the tables in a unique .xlsx file with different sheets and then add a caption that explains all the sheets (Tables).
Additionally, Supplementary Table 2 has the name of the sheet which is "Table_S1", please correct it and also indicate which threshold you used when the Wilcoxon test was performed and what the data on the columns mean.
The same for Supplementary Table 3, in which the name sheet is mislabelled “Table_S2_LEF1_TCGA_survival” and there is no caption.

Corrected as suggested.

Please adjust the references according to the PLOS ONE format.

Done.
